# Phenotype and genotype predictors of BMI variability among European adults

**DOI:** 10.1038/s41387-018-0041-1

**Published:** 2018-05-24

**Authors:** Leticia Goni, Marta García-Granero, Fermín I. Milagro, Marta Cuervo, J. Alfredo Martínez

**Affiliations:** 1 Department of Nutrition, Food Sciences and Physiology, Faculty of Pharmacy and Nutrition, University of Navarra, Pamplona, Navarra Spain; 2 Centre for Nutrition Research, Faculty of Pharmacy and Nutrition, University of Navarra, Pamplona, Navarra Spain; 3 Department of Biochemistry and Genetics, Faculty of Sciences, University of Navarra, Pamplona, Navarra Spain; 4 Biomedical Research Centre Network in Physiopathology of Obesity and Nutrition (CIBERobn), Institute of Health Carlos III, Madrid, Spain; 5 Navarra Institute for Health Research (IdiSNA), Pamplona, Navarra Spain

## Abstract

**Background/Objective:**

Obesity is a complex and multifactorial disease resulting from the interactions among genetics, metabolic, behavioral, sociocultural and environmental factors. In this sense, the aim of the present study was to identify phenotype and genotype variables that could be relevant determinants of body mass index (BMI) variability.

**Subjects/Methods:**

In the present study, a total of 1050 subjects (798 females; 76%) were included. Least angle regression (LARS) analysis was used as regression model selection technique, where the dependent variable was BMI and the independent variables were age, sex, energy intake, physical activity level, and 16 polymorphisms previously related to obesity and lipid metabolism.

**Results:**

The LARS analysis obtained the following formula for BMI explanation: (64.7 + 0.10 × age [years] + 0.42 × gender [0, men; 1, women] + −40.6 × physical activity [physical activity level] + 0.004 × energy intake [kcal] + 0.74 × rs9939609 [0 or 1–2 risk alleles] + −0.72 × rs1800206 [0 or 1–2 risk alleles] + −0.86 × rs1801282 [0 or 1–2 risk alleles] + 0.87 × rs429358 [0 or 1–2 risk alleles]. The multivariable regression model accounted for 21% of the phenotypic variance in BMI. The regression model was internally validated by the bootstrap method (*r*^2^ original data set = 0.208, mean *r*^2^ bootstrap data sets = 0.210).

**Conclusion:**

In conclusion, age, physical activity, energy intake and polymorphisms in *FTO*, *APOE*, *PPARG* and *PPARA* genes are significant predictors of the BMI trait.

## Introduction

In the past 50 years, the prevalence of obesity has steadily raised becoming a global public health problem contributing for a huge increase of health-care costs^1^. It has been estimated that 2.16 billion adults will be overweight and 1.12 billion adults will be obese by 2030, if the present trends continue^2^. An increase in the global burden of overweight and obesity will translate into an increase of the risk of several other health conditions, including type 2 diabetes, cardiovascular disease or certain types of cancer^1^. Although obesity is generally attributed to an imbalance between the energy consumed and the energy expenditure, it is also accepted that it is a complex and a multifactorial disease resulting from genetic, physiological, behavioral, sociocultural and environmental factors^3–7^.

Heritability studies indicate that genetic factors could account for 31–90% of the body inter-individual weight variability^8^. However, the large number of single-nucleotide polymorphisms (SNPs) identified by genome-wide association studies (GWAS) and candidate gene studies, appeared to explain only 2–4% of the obesity status^9^. Even taken together such polymorphisms, they seemed to provide very little risk prediction of the disease^10^. In one of the last GWAS related to adiposity, the 97 genome-wide significant loci identified associated with obesity accounted for 2.7% of the body mass index (BMI) variance^11^.

In addition, a limited predictive value of genetic markers have been described, specifically when they are compared to classical non-genetic risk factors^12,13^. In this context, the design and development of a multivariable regression model based on phenotype and genotype variables could lead us toward the development of more effective precision preventive and treatment dietary interventions^14^. Therefore, the aim of the present study was to identify, in an adult population, phenotype and genotype variables, that combined in a multivariable model, could be associated with BMI variability.

## Subjects and methods

### Study population

The data set included men and women of Caucasian ancestry, who voluntarily attended community pharmacies in Spain. Genotype information of 1065 individuals was available. Of these, 7 subjects were excluded due to missing values for dietary intake, physical activity and/or anthropometric measurements, and 8 subjects were removed because they were <18 years old. Therefore, a total of 1050 subjects were included in the present study.

Individuals were specifically asked if they would be willing to take part anonymously in the research study. After ensuring that participants had understood the information, only those who provided written informed consent for participation were enrolled. All procedures followed were in accordance with the ethical standards of the responsible committee on human experimentation and with the Helsinki Declaration of 1975, as revised in 2000. The Research Ethics Committee of the University of Navarra gave confirmation of fulfillment of the ethical standards and deontological criteria affecting the present survey (Ref. 2410/2014).

### Data collection

Anthropometrics, habitual dietary intake and physical activity measurements were collected by trained nutritionists using a standardized protocol previously described^15^. Briefly, weight and height were measured with a digital scale (Tanita BF-522W, Tanita Corporation, Tokyo, Japan) and a portable stadiometer (Leicester Tanita), respectively. BMI was calculated as weight (kg)/height^2^ (m^2^).

Habitual dietary intake was determined using a validated food groups frequency questionnaire, where basic foods were categorized into 19 food groups. Subjects were asked to report how often (daily, weekly, monthly or never) they had consumed a choice of each food group^16^. Physical activity was estimated by a short 24 h physical activity questionnaire in which subjects were asked about the number of hours resting and practicing activities during a weekday and a weekend day^17^.

### DNA isolation and genotyping

Genomic DNA was obtained from oral epithelial cells collected by ORAcollect DNA^®^ (DNAGenotek, Kanata, Ont., Canada). It was isolated by QIAcube using QiAmp DNA Mini QIAcube Kit (Qiagen, Hilden, Germany), following the manufacturer’s procedures. Sixteen polymorphisms previously associated in the scientific database with body weight regulation and lipid metabolism (rs9939609 (*FTO*), rs17782313 (*MC4R*), rs1801133 (*MTHFR*), rs1800206 (*PPARA*), rs1801282 (*PPARG*), rs662799 (*APOA5*), rs429358 (*AAPOE*), rs7412 (*APOE*), rs1800588 (*LIPC*), rs894160 (*PLIN1*), rs1799983 (*NOS3*), rs1260326 (*GCKR*), rs328 (*LPL*), rs12740374 (*CELSR2*), rs1800777 (*CETP*) and rs4939883 (*LIPG*)) were genotyped using Luminex^®^ 100/200TM System (Luminex Corporation, Austin, Texas), which is based on the principles of xMAP^®^ Technology^18–32^. This method uncompressed polystyrene microspheres internally dyed with various ratios of spectrally distinct fluorophores, which are detected by a flow cytometry-based instrument^33^.

### Statistical analyses

Deviation from Hardy–Weinberg equilibrium (HWE) was tested using *χ*^2^ test and allele frequencies were estimated. Least angle regression (LARS) analysis was used as regression model selection technique due to its advantages in speed, interpretability and predictive accuracy^34^. In the current study, the dependent variable was BMI. The independent variables were age, sex, energy intake, physical activity level and the 16 selected polymorphisms. Because LARS algorithm is designed for linear regression with continuous or binary covariates, polymorphisms were recoded in binary variables according to the association between each polymorphism and BMI tested by using dummy linear regression models. In those cases, where there was no significant association and due to the limited frequency of the variant allele, homozygotes of the minor allele (aa) and heterozygotes (Aa) were grouped and compared with major allele homozygotes (AA). Stagewise regression and Lasso were also performed to confirm the selection of the independent variables established by LARS^34^. The independent variables selected by LARS method were combined to generate the regression function. The formula was constructed by adding each genotype or phenotype variable multiplied by its beta coefficient, and the constant of the regression model. To test potential gene–gene and gene–phenotype interactions among the factors selected by LARS, genotype-by-genotype and genotype-by-phenotype product terms were included in the model. Bootstrapping was performed to internally validate the regression model. It was implemented by constructing a number of resamples (*K* = 1000) of the data set that was obtained by random sampling with replacement from the original data set. For multiple comparisons, Benjamini–Hochberg correction was applied. Statistical analyses were performed using Stata SE, version 12.1 (StataCorp, College Station, TX, USA) and R, version 3.3.2 (R Foundation for Statistical Computing, Vienna, Austria). A *p* value of *p* < 0.05 was considered as statistically significant.

## Results

Baseline phenotypic characteristics of the individuals according to gender have been described (Table 1). The genotypes distribution, minor allele frequencies (MAF) and HWE for each polymorphism are listed (Table 2). MAF ranged from 0.02 to 0.45. The distributions of all the polymorphisms alleles were in HWE except the rs1800588 polymorphism located in *LIPC* gene even after Benjamini–Hochberg correction for multiple comparisons.

**Table 1:** Anthropometrical and nutritional characteristics

	Total population (*n* = 1050)	Males (*n* = 252)	Females (*n* = 798)
Age (years)	50.0 (12.8)	47.4 (12.9)	50.8 (12.7)
Height (cm)	164 (9.0)	174 (7.2)	160 (6.6)
Weight (kg)	79.5 (17.1)	91.4 (16.4)	75.7 (15.5)
BMI (kg/m^2^)	29.6 (5.9)	29.9 (5.0)	29.4 (6.1)
*Nutritional status*			
Normal weight (BMI: 18.5–24.9 kg/m^2^)	232 (22.1)	32 (12.7)	200 (25.1)
Overweight (BMI: 25–29.9 kg/m^2^)	377 (35.9)	106 (42.1)	271 (34.0)
Obesity (BMI: ≥30 kg/m^2^) (%)	441 (42.0)	114 (45.2)	327 (41.0)
Energy intake (kcal)	2138 (453)	2438 (492)	2043 (395)
Physical activity level	1.23 (0.03)	1.24 (0.03)	1.23 (0.03)

The *t* test for continuous variables are expressed as medium (s.d.); and *Χ*^2^ test for qualitative variables are expressed as *n* (%)

*BMI* Body mass index

**Table 2:** Genotype, minor allele frequency and Hardy–Weinberg equilibrium calculations of the 16 SNPs included in LARS analysis

Gene	SNP	Major/minor allele	Major allele homozygote (%)	Heterozygote (%)	Minor allele homozygote (%)	MAF	HWE *p* Value
*FTO*	rs9939609	T/A	329 (31.3)	519 (49.4)	202 (19.2)	0.44	0.92
*MC4R*	rs17782313	T/C	646 (61.5)	355 (33.8)	49 (4.7)	0.22	0.98
*MTHFR*	rs1801133	C/T	380 (36.2)	511 (48.7)	159 (15.1)	0.39	0.55
*PPARA*	rs1800206	C/G	881 (83.9)	162 (15.4)	7 (0.7)	0.08	0.88
*PPARG*	rs1801282	C/G	889 (84.7)	152 (14.5)	9 (0.9)	0.08	0.38
*APOA5*	rs662799	T/C	919 (87.5)	126 (12.0)	5 (0.5)	0.06	0.46
*APOE*	rs429358	T/C	842 (80.2)	199 (18.9)	9 (0.9)	0.10	0.76
*APOE*	rs7412	C/T	920 (87.6)	127 (12.1)	3 (0.3)	0.06	0.53
*LIPC*	rs1800588	C/T	617 (58.8)	360 (34.3)	73 (6.9)	0.24	0.04*
*PLIN1*	rs894160	G/A	560 (53.3)	404 (38.5)	86 (8.2)	0.27	0.28
*NOS3*	rs1799983	G/T	418 (39.8)	488 (46.5)	144 (13.7)	0.37	0.93
*GCKR*	rs1260326	C/T	315 (30.0)	527 (50.2)	208 (19.8)	0.45	0.64
*LPL*	rs328	C/G	765 (72.9)	264 (25.1)	21 (2.0)	0.15	0.75
*CELSR2*	rs12740374	G/T	658 (62.3)	250 (33.3)	42 (4.0)	0.21	0.59
*CETP*	rs1800777	G/A	1002 (95.4)	46 (4.4)	2 (0.2)	0.02	0.06
*LIPG*	rs4939883	C/T	756 (72.0)	260 (24.8)	34 (3.2)	0.16	0.05

*MAF* minor allele frequency, *HWE* Hardy–Weinberg equilibrium

*p value < 0.05 after Benjamini–Hochberg correction for multiple comparisons

**Fig. 1:**
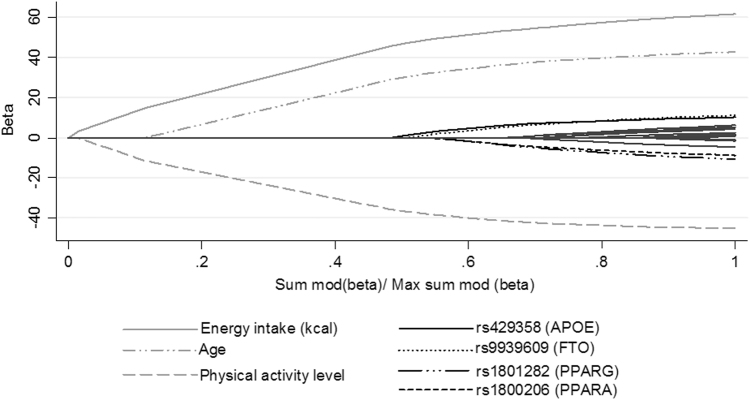
LARS analysis

According to the LARS analysis age, physical activity, energy intake and 4 polymorphisms were associated with BMI variability (Table 3 and Fig. 1). Although gender was not selected by LARS it was included in the model as a common cofounding factor. The LARS analysis obtained the following formula for explain BMI: (64.7 + 0.10 × age [years] + 0.42 × gender [0, men; 1, women] + −40.6 × physical activity [physical activity level] + 0.004 × energy intake [kcal] + 0.74 × rs9939609 [0 or 1–2 risk alleles] + −0.72 × rs1800206 [0 or 1–2 risk alleles] + −0.86 × rs1801282 [0 or 1–2 risk alleles] + 0.87 × rs429358 [0 or 1–2 risk alleles]. The multivariable regression model accounted for 21% of the phenotypic variance in BMI. Energy intake, physical activity level, age, and gender, explained 8.3, 7.3, 4.2 and 0.04%, of the BMI variance, respectively. Among the genotypic variables, *FTO* polymorphism explained 0.1% of the BMI variance, *APOE* polymorphism 0.3%, *PPARG* polymorphism 0.1%, and *PPARA* polymorphism 0.2%. The selection of the independent variables established by LARS was confirmed by stagewise regression and Lasso (data not shown).

**Table 3:** Regression coefficients of the variables selected by LARS

Variables selected	*β*	95% CI	Adjusted *r*^2^	*p* value
Energy intake (kcal)	0.004	0.003;0.005	0.083	<0.001
Physical activity level	−40.6	−49.9;−31.3	0.073	<0.001
Age (years)	0.10	0.08;0.13	0.042	<0.001
rs429358 (*APOE*)^a^	0.86	0.06;1.66	0.003	0.035
rs9939609 (*FTO*)^a^	0.74	0.05;1.42	0.001	0.035
rs1800206 (*PPARA*)^a^	−0.72	−1.58;0.15	0.002	0.104
rs1801282 (*PPARG*)^a^	−0.86	−1.74;0.043	0.001	0.058
Gender	0.42	−0.39;1.24	0.000	0.304
Constant	64.7	43.3;74.9	—	<0.001

^a^Dominant model

Additionally, gene–phenotypic factors and gene–gene interactions were tested. Trend toward significance interactions were found for *FTO* polymorphism and energy intake and for *PPARA* genetic variant and energy intake. When both product terms of the interactions were included in the regression model the adjusted *r*^2^ did not improve significantly (adjusted *r*^2^ for regression model 0.208; adjusted *r*^2^ for the regression model, including interactions 0.212).

**Fig. 2:**
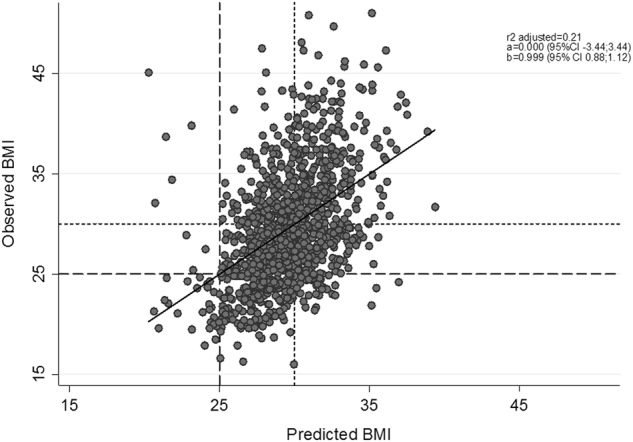
Correlation coefficient between observed BMI and predicted BMI based on the multivariable regression model obtained by LARS

In order to evaluate the accuracy of the model, the relationship between the observed and the predicted BMI was plotted (Fig. 2). The predicted BMI agrees with the observed or “real” BMI by checking the parameters of the linear regression. The intercept of the model is very close to zero and the slope is almost 1, meaning that the change in both variables can be considered proportional.

The internal validation was performed by the bootstrap method, whose estimates agreed closely with the parameters obtained by LARS (*r*^2^ original data set = 0.208, mean *r*^2^ bootstrap data sets = 0.210).

## Discussion

Because common obesity is a multifactorial disease, where genetic, metabolic, physiological, behavioral, sociocultural, and environmental factors are involved, in the current study, a regression model based on phenotype and genotype determinants of BMI has been defined. The regression model includes a total of 4 phenotypic characteristics (age, gender, energy intake, and physical activity) and 4 polymorphisms located next to or in *FTO*, *APOE*, *PPARG*, and *PPARA* genes.

The LARS analysis reported 4 polymorphisms significantly or marginally associated with BMI located in *FTO*, *APOE*, *PPARG*, and *PPARA* genes. *FTO* is a nuclear protein, which is a member of the AlkB related non-haem iron and 2-oxoglutarate-dependent oxygenase superfamily^35^. Although the relationship between *FTO* genetic variant and obesity-related traits (BMI, obesity risk, waist circumference, body fat mass) has been confirmed in several populations, the physiological function of this gene in body weight regulation seems unclear^36,37^.

As far as we know our group reported for the first time, a significant association between rs429358 *APOE* genetic variant and BMI^15^. In the present study, such relationship has been verified in a large sample. The *APOE* gene plays a major role in maintaining plasma lipids homeostasis and it is implicated in adipogenesis^38–40^. *APOE* genetic variants have been associated with several metabolic disorders including high obesity risk^41–44^.

*PPARG* modulates the expression of target genes involved in adipocyte differentiation, insulin sensitivity and inflammatory processes^45,46^, whereas *PPARA* regulates fatty acid oxidation systems^46^. Although in most of candidate gene studies, Pro12Ala has been associated with higher BMI, other authors reported the opposite association or have not found any association at all^23,47–51^. These controversial results suggest that, if this variant does influence obesity predisposition, it may do so through environment-dependent mechanisms. In fact, several studies have reported interactions between *PPARG* and environmental factors such as gender, dietary fat intake, or breast feeding on obesity traits^52–55^. Although the association between genetic variants of the *PPARG* gene and obesity traits has been widely studied, as far as we know there is limited evidence regarding the relationship between *PPARA* variants and obesity phenotype. Meanwhile, Costa-Urrutia et al. (2017) reported a positive association between the rs1800206 *PPARA* polymorphism and obesity risk, Sirbelnagel et al. (2009) did not find a relationship between such genetic variant and BMI or body fat composition^56,57^. We hypothesized that our opposite results regarding *PPARG* and *PPARA* could be due partly to the fact that we have carried out the analysis in the presence of other genetic variants.

Interestingly, 21% of the phenotypic variance in BMI was accounted using the regression model obtained by LARS, including gender, age, energy intake, physical activity and four genetic variants located near or in *FTO*, *APOE*, *PPARG*, and *PPARA* genes. When the polymorphisms were included in the regression model as a genetic risk score, summing the number of risk alleles, the model accounted for 20% of the phenotypic variance. The four polymorphisms accounted for 0.5% of the BMI variability. This finding is in accordance with the studies by Martínez-García et al. (2013), Belsky et al. (2013) and Li et al. (2010), in which a small number of SNPs explained <1% of the BMI heritability^10,12,58^. In this sense, it should be highlighted that when Locke et al. (2015) included a total of 97 SNPs in a prediction model of BMI the authors found a BMI explanation of 2.7%^11^. As far as we know, prediction models that added energy intake and physical activity have not been reported up to date, so we cannot be able to compare our results. However, some authors have observed that, when phenotypical factors are included in the genetic model (such as socioeconomic or depression status), the percentage of the explanation of the BMI significantly increases^10,59^.

Several potential explanations can be offered for the low predictive value of the regression model, but are mainly related to the fact that obesity is characterized for being a multifactorial disease. Although we have included in the model the two main factors that characterized obesity, energy intake and physical activity, there are other features that have not been taken into account such as social determinants (education level, economic status), endocrine disorders (hypothyroidism) or use of certain medications^4,7,60,61^. Another explanation for the low predictive value of the regression model could be related with the marginal effect sizes of the tested variants and the skewed distribution of the effect sizes. In addition, predictive models could include other sources of variation known or hypothesized to influence BMI such as rare variants, gene–gene and gene-environment interactions, copy number variation, and epigenetic and metagenomic effects^14^. Finally, it should be mentioned that in the present study BMI instead of body fat mass was selected as dependent variable. Although BMI is the adiposity measurement most widely used in epidemiological studies, its interpretation does not differ between gender and race, and neither distinguishes between degree of fatness, muscle mass, and skeletal mass^62^. Therefore, it can lead to errors in the estimation of adiposity, over or underestimating adiposity depending on subject complexion; such as athletes or metabolic obese normal weight individuals.

To the best of our knowledge, this is the first study that applies LARS analysis to select phenotype and genotype variables for explain BMI status. However, the study bears some limitations that need to be mentioned. First, the regression model may need to be replicated in an external population. However, the regression model was internally validated by bootstrapping. Second, the present study included only subjects of Caucasian ancestry, so the findings may not be generalizable to other ethnic groups. Third, the model developed in this study used BMI as the response variable instead of body fat mass. Although BMI has some limitations in its interpretability, it is the adiposity measurement more used in epidemiological studies.

In conclusion, significant predictors of BMI included age, energy intake, physical activity, and polymorphisms located near or in *FTO* (rs9939609), *APOE* (rs429358), *PPPARG* (rs1801282), and *PPARA* (rs1800206). Although 4 polymorphisms were selected by LARS, it should be mentioned that they explain a small percentage of BMI variation as has found other authors. Moreover, the proposed statistical method, LARS analysis, could help to implement new criteria for the identification of BMI predictors since obesity is a multifactorial disease in which a large number of phenotypic and genotypic features are involved.
